# A Combined Proteomics and Bioinformatics Approach Reveals Novel Signaling Pathways and Molecular Targets After Intracerebral Hemorrhage

**DOI:** 10.1007/s12031-020-01526-7

**Published:** 2020-03-13

**Authors:** Rajaneekar Dasari, Wenbo Zhi, Frederick Bonsack, Sangeetha Sukumari-Ramesh

**Affiliations:** 1 Department of Pharmacology and Toxicology, Medical College of Georgia, Augusta University, 1120 15th Street, CB3618, Augusta, GA 30912 USA; 2 Center for Biotechnology and Genomic Medicine, Medical College of Georgia, Augusta University, Augusta, USA; 3 Department of Obstetrics and Gynecology, Medical College of Georgia, Augusta University, Augusta, USA

**Keywords:** ICH, Mass spectrometry, Proteomics, Bioinformatics

## Abstract

Intracerebral hemorrhage (ICH) is a non-traumatic cerebrovascular disorder with very high morbidity and mortality and regarded as one of the deadliest stroke subtypes. Notably, there is no effective treatment for ICH. Despite an overall increase in preclinical studies, the pathophysiology of ICH is complex and remains enigmatic. To this end, ICH was induced in male CD-1 mice and the ipsilateral brain tissue was characterized in an unbiased manner using a combination of proteomics and bioinformatics approaches. A total of 4833 proteins were revealed by quantitative proteomic analysis. Of those, 207 proteins exhibited significantly altered expression after ICH in comparison to sham. It was found that 46 proteins were significantly upregulated and 161 proteins were significantly downregulated after ICH compared to sham. The quantitative proteomics approach combined with bioinformatics revealed several novel molecular targets (cyclin-dependent-like kinase 5, E3 ubiquitin-protein ligase, protein phosphatase 2A-alpha, protein phosphatase 2A-beta, serine/threonine-protein kinase PAK1, alpha-actinin-4, calpain-8, axin-1, NCK1, and septin-4), and related signaling pathways, which could play roles in secondary brain injury and long-term neurobehavioral outcomes after ICH warranting further investigation.

**Electronic supplementary material:**

The online version of this article (10.1007/s12031-020-01526-7) contains supplementary material, which is available to authorized users.

## Introduction

Intracerebral hemorrhage (ICH) is defined as non-traumatic extravasation of blood into the brain parenchyma. ICH is the second most common subtype of stroke, accounting for approximately 10–20% of all types of strokes (Feigin et al. 2009; Sacco et al. 2009). Notably, ICH is associated with very high morbidity and mortality and regarded as one of the deadliest stroke subtypes. Around 5.3 million cases of ICH were reported worldwide in 2010, and the ICH-related fatality rate ranges from 35% at 7 days to 59% at 1 year (Hemphill et al. 2015; Rincon and Mayer 2013; Sacco et al. 2009). In addition, survivors of ICH are often left with lifelong disabilities, further implicating the devastating nature of the disease.

The pathophysiological processes following ICH involve both primary and secondary brain injury. The primary brain injury generally occurs within minutes to hours of initial cerebral bleeding and is caused by the mass effect of hematoma whereas the secondary brain injury, resulting from inflammation, oxidative stress, excitotoxicity, and blood-induced cytotoxicity persists for a longer period of time and is mostly responsible for both acute as well as long-term neurological deficits.

Importantly, there is no efficient therapy for ICH, and the clinical management of ICH is largely restricted to supportive care. Lack of treatment options reflects on the complexity as well as the poorly defined pathophysiology of ICH warranting further investigation. To this end, herein, we employ a quantitative proteomics approach combined with bioinformatics in search for novel molecular targets and signaling pathways following ICH. Of note, very few studies, to date, have employed a proteomic approach in elucidating the pathobiology of ICH. More importantly, we isolated single-cell suspensions of brain tissue, which enables the characterization of changes that occur at the cellular level after the hemorrhagic brain injury, and that also limits the extracellular protein-mediated masking of signals derived from low-abundant proteins during the proteomic analysis.

## Materials and Methods

### Induction of ICH

All animal studies were performed according to protocols approved by the Institutional Animal Care and Use Committee in accordance with the NIH and USDA guidelines. As reported previously (Bonsack et al. 2016; Sukumari-Ramesh et al. 2012a, b), ICH was induced in adult male CD-1 mice (8–12 weeks, Charles River, USA). Briefly, mice were anesthetized with ketamine and xylazine and positioned prone on a stereotaxic frame (Stoelting, WI, USA.). Throughout the surgical procedure, the body temperature was maintained at 37 ± 0.5 °C employing a temperature controller (David Kopf Instruments, USA). Using a dental drill (Dremel, USA), a burr hole (0.5 mm) was made 2.2 mm lateral to bregma, not damaging the underlying dura. A Hamilton syringe (26-G) containing 0.04 U of bacterial type IV collagenase (Sigma, USA) in 0.5 μL phosphate-buffered saline (PBS, pH 7.4) was inserted stereotaxically into the left striatum to induce spontaneous ICH. After the needle was removed, the burr hole was covered with bone wax and the incision was sutured. Sham mice underwent the same surgical procedure, but only PBS (0.5 μL) was injected.

### Magnetic Resonance Imaging

Mice were anesthetized with isoflurane (2.5% for induction, 0.7% to 1.5% for maintenance mixed with O_2_). The body temperature was maintained by a water-heated pad and both respiration rate and temperature were monitored and controlled throughout the procedure. Magnetic resonance imaging (MRI) was obtained with a 7 Tesla small animal imaging system from Bruker (Bruker Biospin USR 70/20) using a transmit-receive surface coil. Mice were imaged using a dedicated mouse table fitted with holders to minimize movement during the imaging procedure. After positioning using a triplanar FLASH sequence, MRI studies were performed using susceptibility-weighted sequences with parameters TR = 624.7 ms, TE = 15 ms, 35° flip angle, 24 × 24 FOV, 256 × 256 matrix, 20 slices with a slice thickness of 0.5 mm, and NEX = 2, and T2-weighted MR images were obtained with parameters, TR = 2500 ms, TE = 35 ms, 24 × 24 FOV, 256 × 256 matrix, and 20 slices with a slice thickness of 0.5 mm.

### Neurological Evaluation

Mice were evaluated for neurobehavioral deficits, as per our laboratory and others (Clark et al. 1998; Rosenberg et al. 1990; Sukumari-Ramesh and Alleyne 2016). The neurological deficit was estimated on a 24-point scale and the neurobehavioral analysis was comprised of 6 different tests; circling, climbing, beam walking, compulsory circling, bilateral grasp, and whisker response. Each test was graded from 0 (performance without impairment) to 4 (severe impairment) and a composite neurological deficit score was calculated as the sum of the scores on all the six tests, with a maximum neurological deficit score of 24.

### Sample Preparation for LC-MS/MS (Liquid Chromatography-Mass Spectrometry/Mass Spectrometry) Analysis

Mice were deeply anesthetized and perfused transcardially with ice-cold PBS. The ipsilateral hemisphere containing both hematoma and perihematomal brain tissue was collected from the ICH animals. The respective brain area from sham animals served as the control. To study the proteomic changes that occur at the cellular level, single-cell suspensions were prepared by dissociating the brain tissue by passing through a 100-μm cell strainer using the plunger end of a 1-ml syringe and centrifuged at 1200 rpm for 5 min at 4 °C. The resultant pellet was sonicated and centrifuged at 14,000 rpm for 5 min at 4 °C and the supernatant was collected for LC-MS/MS analysis and using BCA protein assay kit (Pierce, Rockford, IL, USA) the protein concentration was estimated. One hundred micrograms of protein was reduced with dithiothreitol, alkylated using iodoacetamide, and digested overnight using sequencing-grade trypsin (catalogue number: PI90305, Thermo Scientific, USA). Digested peptides were cleaned up using C18 spin column (catalogue number: 74-4607, Harvard Apparatus, USA) and then lyophilized.

### LC-MS/MS Analysis

Peptide digests were analyzed on an Orbitrap Fusion tribrid mass spectrometer (Thermo Scientific, USA) coupled with an Ultimate 3000 nano-UPLC (ultra-performance liquid chromatography) system (Thermo Scientific, USA). Two microliters of reconstituted peptide was first trapped and washed on a Pepmap100 C18 trap (5 μm, 0.3 × 5mm) at 20 μl/min using 2% acetonitrile in water (with 0.1% formic acid) for 10 min and then separated on a Pepman 100 rapid-separation liquid chromatography (RSLC) C18 column (2.0 μm, 75-μm × 150-mm) using a gradient of 2 to 40% acetonitrile with 0.1% formic acid over 100 min at a flow rate of 300 nl/min and a column temperature of 40 °C.

Samples were analyzed by data-dependent acquisition in positive mode using Orbitrap MS analyzer for precursor scan at 120,000 FWHM (full width at half maximum) from 300 to 1500 m/z and ion-trap MS analyzer for MS/MS scans at top speed mode (3-s cycle time). Collision-induced dissociation (CID) was used as a fragmentation method. Raw MS data were processed using Proteome Discoverer (v1.4, Thermo Scientific, USA) and submitted for the Sequest HT database searching algorithm against the Uniprot mouse database. The percolator PSM (peptide spectrum match) validator algorithm was used for peptide spectrum matching validation. SequestHT search parameters included 10 ppm precursor and 0.6 Da product ion mass tolerance, with static Carbamidomethylation of cysteine and dynamic oxidation of methionine.

### Tissue Preparation and Western Blotting

For western blotting, the brain tissue samples in RIPA buffer containing protease and phosphatase inhibitors were centrifuged at 14,000 rpm for 5 min and the supernatant was collected. The protein concentrations were estimated by using BCA protein assay kit (Pierce, Rockford, IL, USA). Fifty micrograms of protein were separated on a 4–20% sodium dodecyl sulfate-polyacrylamide gel and transferred on to a polyvinylidene fluoride (PVDF) membrane. By using 5% non-fat dry milk, the membrane was blocked. Then, the blots were incubated with appropriate primary antibody overnight at 4 °C and followed by 2 h incubation by fluorescent-tagged secondary antibody at room temperature. Using a Li-Cor Odyssey near infrared imaging system, as previously reported by our group (Sukumari-Ramesh et al. 2016; Sukumari-Ramesh et al. 2010, 2011; Wakade et al. 2010), the blots were visualized. The densitometry analysis was performed using Quantity One software (Bio-Rad, USA), as reported earlier (Sukumari-Ramesh et al. 2016, 2010, 2011; Wakade et al. 2010). Briefly, the pixel identity of the protein band and the background was estimated, and background correction was done by background subtraction. The data was normalized with β-Actin, which served as the loading control and data was analyzed using GraphPad Prism 8 software (GraphPad Software Inc., USA).

### Statistical Analysis

For quantitative LC-MS/MS analysis, the peptide spectrum match (PSM) count for each identified protein was used as a semi-quantitative measure for protein level. The PSM count for each protein in a specific sample was normalized using the total PSM counts for that sample to compensate for possible sample loading variations. After normalization across all samples, the mean PSM count for the three replicates is calculated for each protein and each sample group. The average PSM counts for each sample group were used for statistical analysis with Microsoft Excel (Microsoft Corporation, USA). The differences between the two groups were assessed by Student’s *t* test. A *p* < 0.05 was considered to be statistically significant.

## Results

### Mass Spectrometry-Based Identification of Differentially Expressed Proteins After ICH

**Fig. 1:**
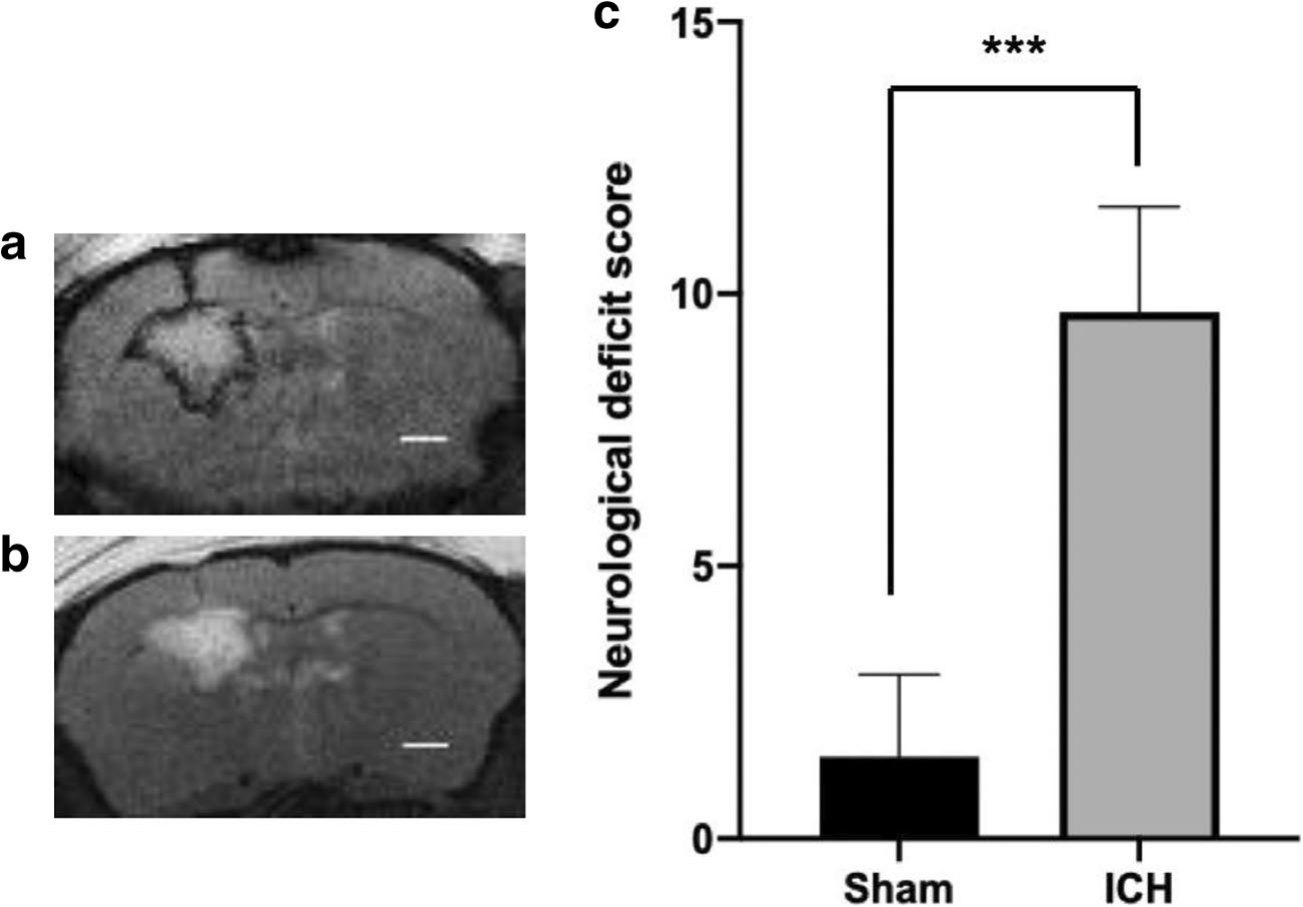
ICH was induced in male CD1 mice, as detailed in the “Methods” section, and the susceptibility-weighted (**a**), as well as T2-weighted (**b**), magnetic resonance (MR) image depicts ICH-mediated induction of hematoma as well as the hemorrhagic lesion, respectively, in the mouse brain striatum on day 3 post-surgery (scale bar = 1 mm). The induction of ICH resulted in significant neurobehavioral deficits, which was estimated using a 24-point scale as detailed in methods, on day 3 post-surgery in comparison to sham that served as the experimental control (**c**). *n* = 8–9/animals per group. ***p < 0.001 vs. sham

ICH was induced in the striatum of male CD1 mice using collagenase injection method. Induction of ICH resulted in a hematoma as well as hemorrhagic lesion, as demonstrated by susceptibility-weighted as well as T2-weighted magnetic resonance (MR) image, respectively (Fig. 1a, b). For proteomic analysis employing mass spectrometry, we collected both hematomal and perihematomal ipsilateral brain tissue from ICH animals on day 3 post-injury, an acute time point which exhibited prominent induction of both microglial and astrocyte activation (Bonsack et al. 2016; Sukumari-Ramesh et al. 2012a, b), critical brain responses that contribute to secondary brain damage and brain recovery after ICH. Of note, single-cell suspensions of brain tissue were isolated, as described in methods and subjected to LC-MS/MS analysis. The striatal brain area from sham animals served as the experimental control. Soon before the collection of brain samples for proteomic studies, the animals were subjected to neurobehavioral analysis to confirm the induction of ICH. Notably, ICH animals exhibited very profound neurobehavioral deficits in comparison to sham (*p* < 0.001) (Fig. 1c).

**Fig. 2:**
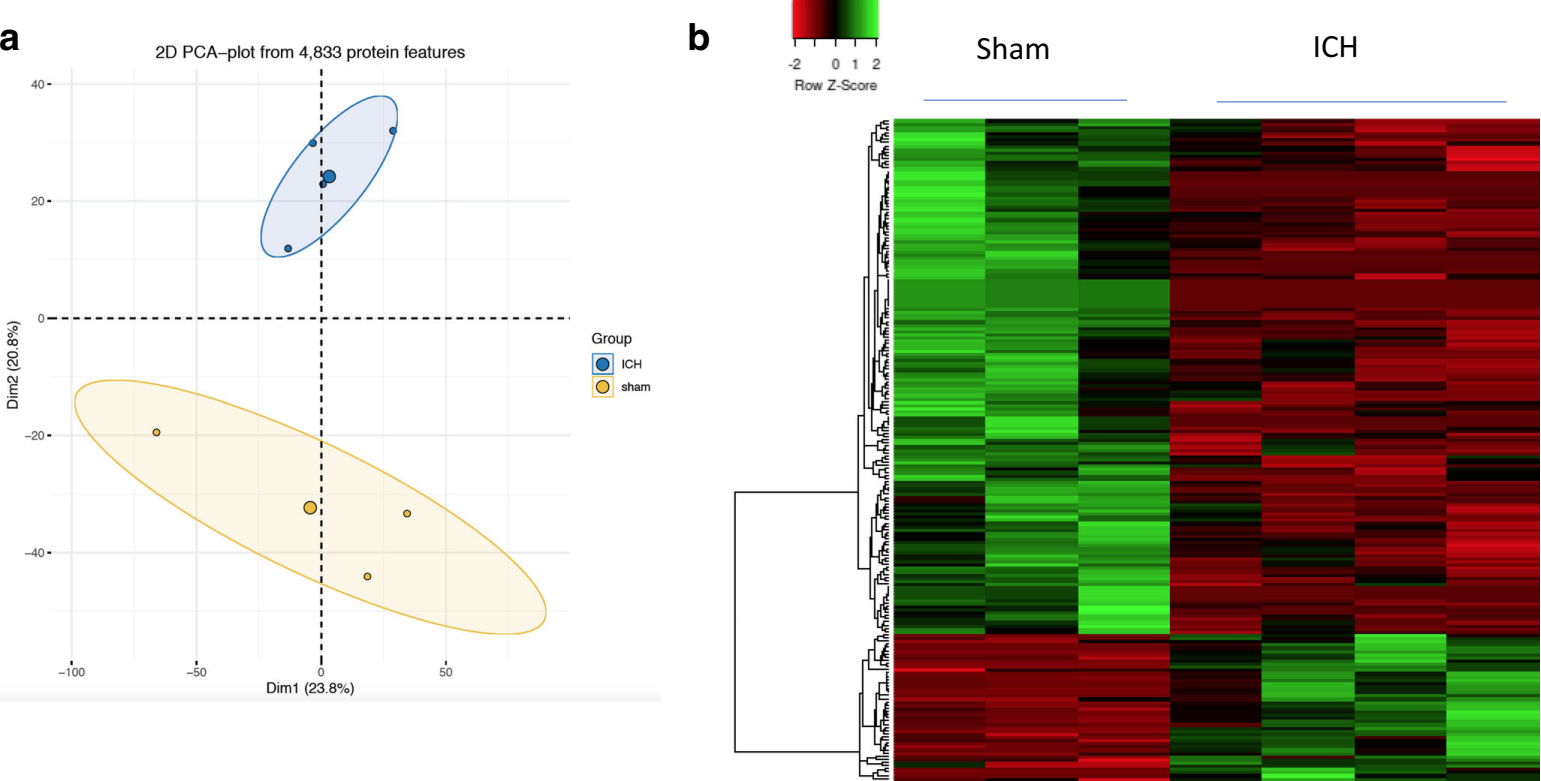
The principal component analysis was performed with normalized PSM counts of proteins identified by mass spectrometry using the statistical computing software R and it illustrates that the replicates in each experimental group (sham/ICH) are closely clustered (**a**). Heatmap representation of differentially expressed proteins between sham and ICH (**b**). The heatmap was generated with normalized PSM counts of differentially expressed proteins using a web server, heatmapper available at http://www.heatmapper.ca using the distance function (pearson) and clustering (average linkage). Forty-six proteins were significantly upregulated and 161 proteins were significantly downregulated after ICH in comparison to sham. Green: high expression, and red: low expression. *n* = 3–4 animals/group. The complete list of differentially expressed proteins is provided as supplementary data Table 1

**Fig. 3:**
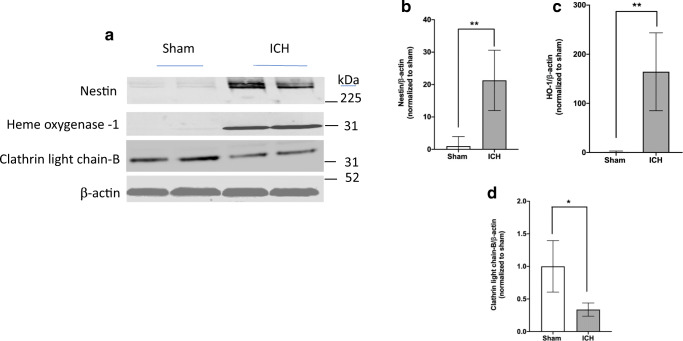
Validation of proteomic analysis. To validate the proteomic analysis, western blotting was performed and analyzed the expression of proteins, nestin, heme oxygenase-1 (HO-1), and clathrin light chain B after sham or ICH, as detailed in methods (**a**). Consistent with the proteomic studies, the densitometry analysis of the western blotting data demonstrated significant upregulation of nestin (**b**) as well as heme oxygenase-1 (HO-1) (**c**) and significant downregulation of clathrin light chain B (**d**) after ICH in comparison to sham. β-actin was used as a loading control (** *p* < 0.01, * *p* < 0.05 vs. sham). *n* = 4 animals/group

Initial proteomic analysis revealed the expression of 4833 proteins between sham and ICH and the principal component analysis differentiated the proteomic profile between sham and ICH groups (Fig. 2a). Of note, 207 proteins exhibited a significant difference in their expression profile between sham and ICH, on the basis of quantitative proteomic analysis. Precisely, 46 proteins were found to be significantly upregulated, and 161 were significantly downregulated after ICH in comparison to sham (Fig. 2b, Supplementary data Table 1). To validate the data, three proteins were randomly selected and their expression changes were analyzed using western blotting. Consistent with the mass spectrometry analysis, nestin and heme oxygenase 1 were found to be upregulated after ICH, whereas clathrin light chain B was found to be downregulated after ICH in comparison to sham (Fig. 3) ascertaining the efficacy of the proteomic approach.

### Functional Characterization of Differentially Expressed Proteins by Bioinformatics Approach

**Fig. 4:**
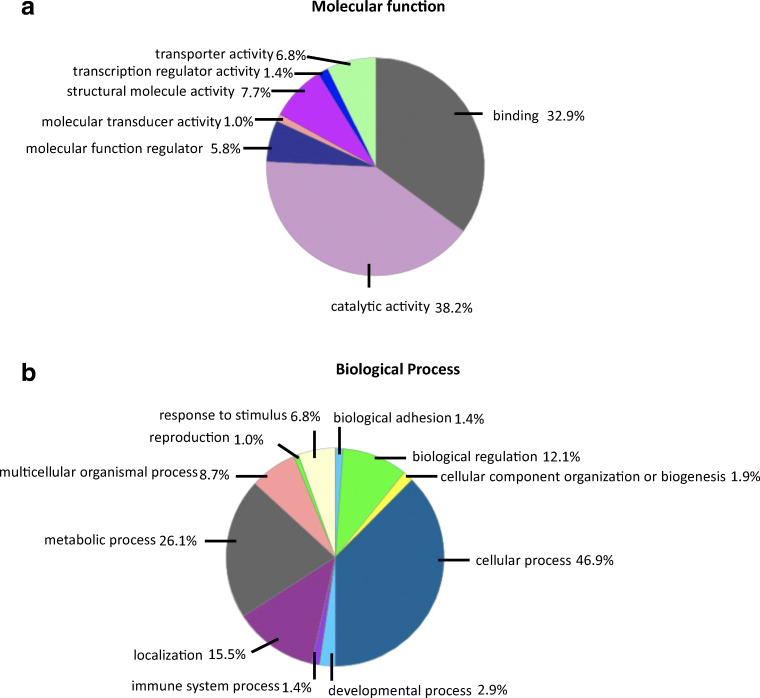

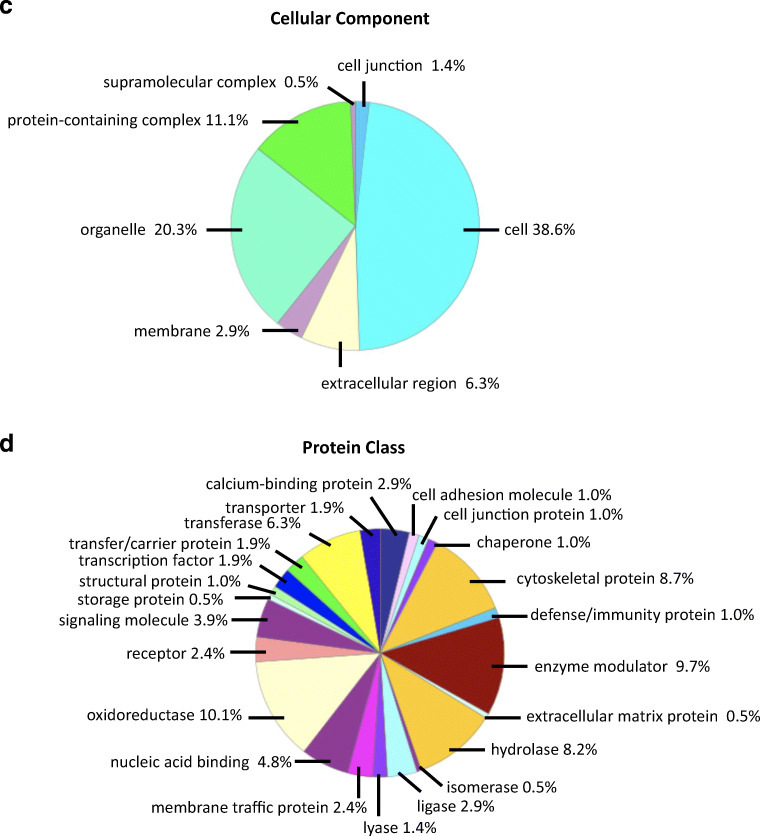
Gene ontology analysis of the differentially expressed proteins between sham and ICH employing bioinformatics application, PANTHER. The analysis resulted in the categorization of proteins into various groups based on molecular function (**a**), biological process (**b**), cellular components (**c**), and protein class (**d**). The classification of differentially expressed proteins based on protein class is provided as supplementary data Table 2

To gain insight into the functional roles of differentially expressed proteins as well as the associated signaling pathways, we analyzed the data derived from the proteomic analysis using a bioinformatics application, PANTHER (Mi et al., 2019), against the mice protein ontology database using UniPort accession number. The bioinformatics analysis resulted in the categorization of proteins into various groups based on molecular function, biological process, cellular components, protein class, and signaling pathways. As per the analysis, the prominent molecular functions that the proteins carried were of catalytic activity (38.2%) and binding (32.9%), (Fig. 4a), and the major biological processes that the proteins involved were of cellular process (46.9%), metabolic process (26.1%), localization (15.5%), and biological regulation (12.1%) (Fig. 4b). Also, most of the differentially expressed proteins were derived from cell (38.6%) and organelle (20.3%) (Fig. 4c) and based on protein class, the majority of the proteins belonged to oxidoreductase (10.1%), enzyme modulators (9.7%), cytoskeletal proteins (8.7%), hydrolases (8.2%), and transferase (6.3%) (Fig. 4d, Supplementary data Table 2).

### Identification of Molecular Pathways and Associated Proteins in Relation to ICH

**Fig. 5:**
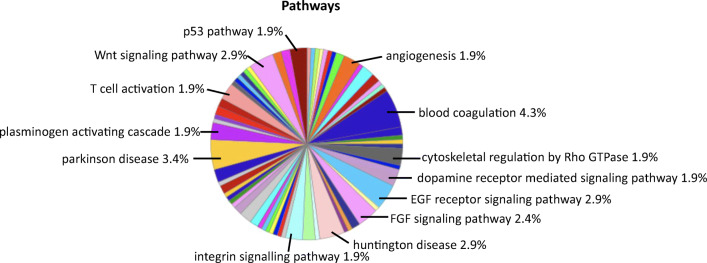
Bioinformatics analysis of differentially expressed proteins based on signaling pathways. The prominent signaling pathways are indicated and the complete list of pathways associated with the differentially expressed proteins is provided as supplementary data Table 3

**Fig. 6:**
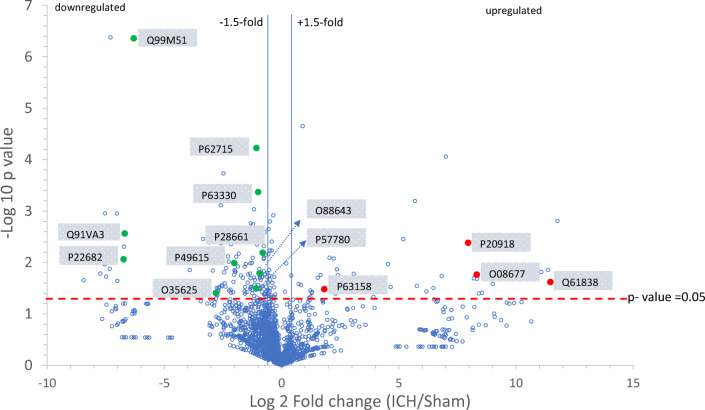
The volcano plot of the entire proteome (sham and ICH) identified by mass spectrometry demonstrates the protein distribution on which proteins with statistically significant differential expression (*p* < 0.05) are located above the horizontal line (*p* value =0 .05) and the vertical lines denote a fold change of approximately ± 1.5. The key proteins associated with the identified pathways are labeled

Further, the PANTHER-based analysis of proteomic data identified 67 different molecular pathways, and of those, the key pathways which were modulated between ICH and sham included blood coagulation, plasminogen activating cascade, p53 pathway, EGF receptor signaling pathway, FGF signaling pathway, cytoskeletal regulation by Rho GTPase, angiogenesis, integrin signaling pathway, Parkinson’s disease, Huntington disease, dopamine receptor mediated signaling pathway, T cell activation, and Wnt signaling (Fig. 5 and Supplementary data Table 3). Upon further evaluation employing PANTHER, the key proteins that were associated with the signaling cascades were alpha-2-macroglobulin, kininogen-1, plasminogen, high mobility group protein B1 (HMGB1), cyclin-dependent-like kinase 5, E3 ubiquitin-protein ligase, protein phosphatase 2A-alpha, protein phosphatase 2A-beta, serine/threonine-protein kinase PAK1, alpha-actinin-4, calpain-8, axin-1, NCK1 (non-catalytic region of tyrosine kinase adaptor protein 1), and septin-4 (Table 1 and Fig. 6). Notably, proteins such as alpha-2-macroglobulin, kininogen-1, plasminogen, and HMGB1were found to be upregulated after ICH in comparison to sham (*p* < 0.05) whereas cyclin-dependent-like kinase 5, E3 ubiquitin-protein ligase, protein phosphatase 2A-alpha, protein phosphatase 2A-beta, serine/threonine-protein kinase PAK1, alpha-actinin-4, calpain-8, axin-1, NCK1 (non-catalytic region of tyrosine kinase adaptor protein 1), and septin-4 were found to be downregulated after ICH in comparison to sham (*p* < 0.05) (Table 1).

**Table 1:** Major signaling pathways associated with the differentially expressed proteins between sham and ICH. The description of key proteins associated with the pathways and ICH-induced protein modulation in comparison to sham is provided

Key proteins associated with the identified pathways				
Signaling pathway	Associated protein	Accession number	Brain injury-induced modulation Upregulation↑ or downregulation↓	*p* value
Blood coagulation				
	Alpha-2-macroglobulin	Q61838	↑	0.023875
	Kininogen-1	O08677	↑	0.017174
	Plasminogen	P20918	↑	0.004127
Plasminogen activating cascade				
	Plasminogen	P20918	↑	0.004127
P53 pathway				
	High mobility group protein B1 (HMGB1)	P63158	↑	0.032999
	Cyclin-dependent-like kinase 5	P49615	↓	0.010177
EGF receptor signaling pathway				
	E3 ubiquitin-protein ligase CBL	P22682	↓	0.008644
FGF signaling pathway				
	Protein phosphatase 2A-alpha	P63330	↓	0.000424
	Protein phosphatase 2A-beta	P62715	↓	0.0000596
Cytoskeletal regulation by Rho GTPase				
	Serine/threonine-protein kinase PAK1	O88643	↓	0.016255
Angiogenesis				
	Axin 1	O35625	↓	0.039058
	NCK1	Q99M51	↓	0.000000436
Integrin signaling pathway				
	Alpha-actinin-4	P57780	↓	0.032023
Parkinson’s disease				
	Septin-4	P28661	↓	0.006433
Huntington Disease				
	Calpain-8	Q91VA3	↓	0.002722
Dopamine receptor mediated signaling pathway				
	Cyclin-dependent-like kinase 5	P49615	↓	0.010177
T cell activation				
	NCK1	Q99M51	↓	0.000000436
Wnt signaling pathway				
	Axin-1	O35625	↓	0.039058

## Discussion

Intracerebral hemorrhage is a fatal stroke subtype and major public health problem. Importantly, the mortality of ICH has not changed over the past 20 years (Flaherty et al. 2006). Also, approximately 80% of survivors of ICH are often left with neurobehavioral disabilities (Agnihotri et al. 2011). Given the fact that advanced age is a risk factor of ICH (van Asch et al. 2010), the incidence of ICH is expected to increase over the next decades due to possible changes in demographics. Notably, the pathophysiology of ICH remains largely undefined.

Herein, we employed a comprehensive proteomics approach combined with bioinformatics in characterizing the molecular changes in the ipsilateral brain region at the cellular level following ICH. Using this method, we identified that the differentially expressed proteins between sham and ICH were associated with 13 prominent signaling pathways, which were blood coagulation, plasminogen activating cascade, p53 pathway, EGF receptor signaling pathway, FGF signaling pathway, cytoskeletal regulation by Rho GTPase, angiogenesis, integrin signaling pathway, Parkinson’s disease, Huntington disease, dopamine receptor mediated signaling pathway, T cell activation, and Wnt signaling.

Blood coagulation involves a sequential conversion of a series of proenzymes to functionally active proteases (Lee et al. 1996). As a consequence of ICH, blood accumulates in the brain tissue surrounding the blood vessel rupture leading to the formation of a hematoma. Cerebral hemorrhage activates blood coagulation cascade, which in turn results in the release and subsequent brain accumulation of large amounts of thrombin, a proteolytic enzyme that plays a critical role in ICH-induced perihematomal edema, a contributing factor in delayed neurologic deterioration. To this end, the mass spectrometry analysis revealed augmented expression of alpha-2-macroglobulin, a thrombin/protease inhibitor, in ICH samples compared to sham. Elevated levels of serum protein, alpha-2-macroglobulin after ICH could be due to ICH-induced damage of blood-brain barrier and subsequent accumulation of alpha-2-macroglobulin in the brain. However, the role of alpha-2-macroglobulin in modulating the proteolytic activity of thrombin and thereby ICH-induced brain injury is yet to be defined. Also, a serum protein that plays a key role in fibrinolysis, plasminogen, was found to be higher after ICH in comparison to sham and bioinformatics analysis also identified plasminogen activating cascade as a key signaling pathway after ICH. In addition, another blood coagulation pathway-related protein that was identified by the proteomic approach was kininogen-1. High-molecular-weight kininogen is an important constituent of the plasma contact-kinin system, which represents a network of serially connected serine proteases. Though kininogen appears to be a critical regulator of thrombus formation and inflammation after brain ischemia (Langhauser et al., 2012), its functional role remains largely unexplored after ICH.

ICH results in the brain accumulation of various blood components such as hemoglobin and its degradation products such as hemin, which play critical roles in secondary brain damage and neurological deficits (Madangarli et al., 2019). Consistently, hemoglobin (Hb) subunit alpha, Hb subunit beta, Hb subunit epsilon and ferritin light chain (a subunit of iron storage protein ferritin), and a heme catabolizing enzyme, heme oxygenase-1, were found to be elevated in the brain of ICH animals, in comparison to sham by mass spectrometry analysis. In addition, the complement components which enter the brain tissue following ICH (Cao et al. 2016; Hua et al. 2000) initiate complement-mediated brain damage by forming the membrane attack complex (MAC) which in turn causes erythrocyte lysis, induces neuronal death, and modulates cellular pathways related to cytokines and reactive oxygen species (Ducruet et al. 2009; Hua et al. 2000). Along these lines, increased expression of complement component-3 was observed after ICH in comparison to sham by mass spectrometry. Of note, various blood components play critical roles in ICH-induced inflammatory and oxidative brain damage. Consistently, proinflammatory molecule, HMGB1 was found to be upregulated after ICH in comparison to sham. Notably, bioinformatics analysis identified HMGB1 as a protein involved in the p53 pathway, which is in line with the reports, which revealed that HMGB1 can regulate DNA binding of p53 (Jayaraman et al. 1998; Rowell et al. 2012). p53 signaling plays a critical role in hemin-induced neuronal apoptosis after ICH (Wang et al. 2017). However, the functional link between p53 signaling and HMGB1 is largely unknown after ICH requiring investigation.

The epidermal growth factor (EGF), a neurotrophic factor (Yamada et al. 1997), is a key regulator of neural progenitor cell proliferation (Pillai et al. 2013). Thus, EGF could play roles in post-stroke recovery. EGF can also modulate blood-brain barrier (BBB) permeability by activating EGF receptor (EGFR) and mitogen-activated protein kinase (MAPK) signaling pathway (Chen et al. 2015; Liu et al. 2014). Of note, BBB destruction is a hallmark of ICH (Keep et al. 2008) and plays a crucial role in ICH-induced secondary brain damage. Apart from a recent study, which demonstrated that administration of gelatin hydrogel containing epidermal growth factor improved neurological recovery after intracerebral hemorrhage (ICH) (Lim et al. 2019), the functional significance of EGFR signaling, as well as its key regulator such as E3 ubiquitin-protein ligase (Huangfu and Fuchs 2010), remains least explored after ICH.

The fibroblast growth factor (FGF) plays an important role in the regulation of vascular integrity (Murakami et al. 2008) as well as astrogliosis (Kang et al. 2014), the critical brain response to brain injury that plays roles in both brain damage as well as brain recovery. Consistently, it has been documented that exogenous FGF treatment preserved BBB integrity after ICH (Huang et al., 2012). Our proteomic analysis revealed ICH-induced modulation of the alpha and beta subunits of protein phosphatase 2A, which is a downstream regulator of FGF signaling. However, the functional role of protein phosphatase 2A after ICH is yet to be studied.

Pak1 (p21-activated serine/threonine kinase 1), a downstream effector of Rho GTPases, regulates a variety of cellular processes by remodeling the cytoskeleton and by promoting gene transcription and cell survival. Pak1 is involved in synaptic strength (Gonzalez-Forero et al., 2012), and impairment in PAK pathways could result in neurodegeneration. Downregulation of PAK signaling is implicated in neurodegenerative diseases such as Alzheimer’s disease and Huntington disease (Ma et al. 2012). Herein, we document the downregulation of Pak1 after ICH in comparison to sham, based on the quantitative proteomic analysis. However, the functional significance of Pak1 remains largely unexplored after ICH.

The Wnt pathway is an evolutionarily conserved signaling pathway that plays a role in blood-brain barrier dysfunction, secondary brain damage, and angiogenesis after ICH (Li et al. 2017; Wang et al. 2019; Zhao et al. 2017). Importantly, the proteomic analysis revealed ICH- induced downregulation of a protein, axin-1, which is a key regulator of Wnt signaling (Jiang et al. 2018). However, the functional role of axin-1, as well as the mechanism by which axin-1 regulates secondary brain damage or angiogenesis after ICH, is yet to be explored.

Integrins are a major family of cell adhesion molecules that play critical role in angiogenesis (Brooks 1996; Silva et al. 2008; Stromblad and Cheresh 1996; Stupack and Cheresh 2004; Westlin 2001), vascular integrity (Stromblad and Cheresh 1996), and inflammatory responses (Schittenhelm et al. 2017). However, the functional role of integrin signaling remains least explored after ICH requiring investigation.

Lymphocytes are detected in the perihematomal brain tissue of ICH patients (Guo et al. 2006). Also, in animal models of ICH, the infiltration of T cells occurs after ICH (Loftspring et al. 2009; Xue and Del Bigio 2000, 2003) and the predominant T cell after ICH is CD4+ (Mracsko et al. 2014). Another T cell population observed after ICH is anti-inflammatory, T regulatory cells (Treg) and adoptive transfer of Treg improved neurological outcomes after ICH (Yang et al. 2014). However, compared to ischemic stroke, T cell activation is least characterized after ICH warranting investigation.

Based on the quantitative proteomic analysis, we found the downregulation of protein, septin 4 after ICH, in comparison to control. Septins are proteins involved in the regulation of synaptic vesicle trafficking and neurotransmitter release. Importantly, mice lacking presynaptic protein, Septin 4, exhibited diminished dopaminergic neurotransmission compared to control and septin-4 contributes to alpha-synuclein-induced neurotoxicity (Ihara et al. 2007) implicating the need to explore the relevance of ICH-induced septin-4 downregulation. Further, though the frequent location of the occurrence of ICH in humans is basal ganglia (Zuo et al. 2017), a brain region that plays a critical role in Parkinson’s disease (PD), whether ICH increases the risk of PD remains largely unknown and it requires investigation. Also, the bioinformatics studies revealed ICH-induced downregulation of protein, cyclin-dependent-like kinase-5, which plays a role in dopaminergic signaling, but its functional role after ICH is yet to be defined. Similarly, based on our proteomic studies, it would be worth investigating whether ICH could increase the risk of developing Huntington disease, a neurogenerative disorder.

Altogether, the present article identifies several molecular targets for the first-time after ICH, which could play critical roles in secondary brain injury and long-term neurobehavioral outcomes. Of note, further studies are warranted characterizing the functional significance of identified candidate proteins in the pathophysiology of ICH, one of the deadliest stroke subtypes.

## Electronic Supplementary Material

ESM 1 (PDF 261 kb)

ESM 2 (DOCX 23 kb)

ESM 3 (DOCX 22 kb)

## Abbreviations

ICH: intracerebral hemorrhage
PBS: phosphate-buffered saline
LC-MS/MS: liquid chromatography-mass spectrometry/mass spectrometry
UPLC: ultra-performance liquid chromatography
RSLC: rapid-separation liquid chromatography
FWHM: full width at half maximum
CID: collision-induced dissociation
PSM: peptide spectrum match
RIPA: radioimmunoprecipitation assay
BCA: bicinchoninic acid assay
PVDF: polyvinylidene fluoride
MRI: magnetic resonance imaging
HMGB1: high mobility group protein B1
NCK1: NCK adaptor protein 1
EGF: epidermal growth factor
EGFR: epidermal growth factor receptor
Hb: hemoglobin
HO-1: heme oxygenase-1
MAC: membrane attack complex
BDNF: brain-derived neurotrophic factor
BBB: blood-brain barrier
MAPK: mitogen-activated protein kinase
FGF: fibroblast growth factor
Pak1: p21-activated serine/threonine kinase 1
