# Supplementary material for: A Combined Proteomics and Bioinformatics Approach Reveals Novel Signaling Pathways and Molecular Targets After Intracerebral Hemorrhage
Source: J Mol Neurosci. 2020 Mar 13;70(8):1186–97. doi: 10.1007/s12031-020-01526-7 (PMC7359136; doi:10.1007/s12031-020-01526-7)
Supplement: Supplementary file 1 — (PDF 261 kb) [file 12031_2020_1526_MOESM1_ESM.pdf]

Supplementary data. Table 1

Accession number	Protein description	Sham	ICH	Brain injury-induced modulation Upregulation ↑ or Downregulation ↓
		Average number of total peptides	Average number of total peptides	
Q9D7P6	Iron-sulfur cluster assembly enzyme ISCU, mitochondrial OS=Mus musculus GN=Iscu PE=1 SV=1 - [ISCU_MOUSE]	1.67	0	↓
Q9D855	Cytochrome b-c1 complex subunit 7 OS=Mus musculus GN=Uqcrb PE=1 SV=3 - [QCR7_MOUSE]	1.00	0	↓
Q59J78	Mimitin, mitochondrial OS=Mus musculus GN=Ndufaf2 PE=1 SV=1 - [MIMIT_MOUSE]	1.00	0	↓
Q8QZR5	Alanine aminotransferase 1 OS=Mus musculus GN=Gpt PE=1 SV=3 - [ALAT1_MOUSE]	1.00	0	↓
Q99M51	Cytoplasmic protein NCK1 OS=Mus musculus GN=Nck1 PE=1 SV=1 - [NCK1_MOUSE]	1.00	0	↓
Q9EP89	Serine beta-lactamase-like protein LACTB, mitochondrial OS=Mus musculus GN=Lactb PE=1 SV=1 - [LACTB_MOUSE]	1.00	0	↓
Q9QX11	Cytohesin-1 OS=Mus musculus GN=Cyth1 PE=1 SV=2 - [CYH1_MOUSE]	1.00	0	↓
Q8BM14	Lipase member K OS=Mus musculus GN=Lipk PE=2 SV=1 - [LIPK_MOUSE]	1.00	0	↓
Q8JZR6	Electroneutral sodium bicarbonate exchanger 1 OS=Mus musculus GN=Slc4a8 PE=2 SV=1 - [S4A8_MOUSE]	1.00	0	↓
P09103	Protein disulfide-isomerase OS=Mus musculus GN=P4hb PE=1 SV=2 - [PDIA1_MOUSE]	8.33	10	↑
P62715	Serine/threonine-protein phosphatase 2A catalytic subunit beta isoform OS=Mus musculus GN=Ppp2cb PE=1 SV=1 - [PP2AB_MOUSE]	9.67	5.5	↓
Q6P5H2	Nestin OS=Mus musculus GN=Nes PE=1 SV=1 - [NEST_MOUSE]	0.00	1	↑
Q9D6J6	NADH dehydrogenase [ubiquinone] flavoprotein 2, mitochondrial OS=Mus musculus GN=Ndufv2 PE=1 SV=2 - [NDUV2_MOUSE]	5.67	1.75	↓
P63330	Serine/threonine-protein phosphatase 2A catalytic subunit alpha isoform OS=Mus musculus GN=Ppp2ca PE=1 SV=1 - [PP2AA_MOUSE]	9.67	5.75	↓
E9PV24	Fibrinogen alpha chain OS=Mus musculus GN=Fga PE=1 SV=1 - [FIBA_MOUSE]	0.33	10	↑
Q8BP47	Asparagine--tRNA ligase, cytoplasmic OS=Mus musculus GN=Nars PE=1 SV=2 - [SYNC_MOUSE]	5.33	1.5	↓
Q9WUA3	ATP-dependent 6-phosphofructokinase, platelet type OS=Mus musculus GN=Pfkp PE=1 SV=1 - [PFKAP_MOUSE]	16.33	10.5	↓
P70297	Signal transducing adapter molecule 1 OS=Mus musculus GN=Stam PE=1 SV=3 - [STAM1_MOUSE]	2.33	0	↓
P43276	Histone H1.5 OS=Mus musculus GN=Hist1h1b PE=1 SV=2 - [H15_MOUSE]	1.33	0	↓
A2AGT5	Cytoskeleton-associated protein 5 OS=Mus musculus GN=Ckap5 PE=1 SV=1 - [CKAP5_MOUSE]	1.67	0	↓
Q3V3V9	Leucine-rich repeat-containing protein 16C OS=Mus musculus GN=Rltpr PE=1 SV=2 - [LR16C_MOUSE]	1.67	0	↓
Q9D1K2	V-type proton ATPase subunit F OS=Mus musculus GN=Atp6v1f PE=1 SV=2 - [VATF_MOUSE]	3.67	3	↓
Q8VCM7	Fibrinogen gamma chain OS=Mus musculus GN=Fgg PE=1 SV=1 - [FIBG_MOUSE]	0.00	8.25	↑
P60879	Synaptosomal-associated protein 25 OS=Mus musculus GN=Snap25 PE=1 SV=1 - [SNP25_MOUSE]	10.33	8.75	↓
Q91VD9	NADH-ubiquinone oxidoreductase 75 kDa subunit, mitochondrial OS=Mus musculus GN=Ndufs1 PE=1 SV=2 - [NDUS1_MOUSE]	13.67	5.75	↓
P28660	Nck-associated protein 1 OS=Mus musculus GN=Nckap1 PE=1 SV=2 - [NCKP1_MOUSE]	7.00	3.25	↓
Q61644	Protein kinase C and casein kinase substrate in neurons protein 1 OS=Mus musculus GN=Pacsin1 PE=1 SV=1 - [PACN1_MOUSE]	14.00	9.5	↓
P19536	Cytochrome c oxidase subunit 5B, mitochondrial OS=Mus musculus GN=Cox5b PE=1 SV=1 - [COX5B_MOUSE]	2.00	1	↓
P28271	Cytoplasmic aconitate hydratase OS=Mus musculus GN=Aco1 PE=1 SV=3 - [ACOC_MOUSE]	3.67	2.25	↓
Q8BIF0	CD99 antigen-like protein 2 OS=Mus musculus GN=Cd99l2 PE=1 SV=1 - [C99L2_MOUSE]	1.33	0	↓
Q3TYX3	SET and MYND domain-containing protein 5 OS=Mus musculus GN=Smyd5 PE=1 SV=2 - [SMYD5_MOUSE]	1.33	0	↓

Continued

Accession number	Protein description	Sham	ICH	Brain injury-induced modulation Upregulation ↑ or Downregulation ↓
		Average number of total peptides	Average number of total peptides	
Q9QWG7	Sulfotransferase family cytosolic 1B member 1 OS=Mus musculus GN=Sult1b1 PE=1 SV=2 - [ST1B1_MOUSE]	1.00	0	↓
Q91VA3	Calpain-8 OS=Mus musculus GN=Capn8 PE=1 SV=1 - [CAN8_MOUSE]	1.00	0	↓
Q80U40	RIMS-binding protein 2 OS=Mus musculus GN=Rimbp2 PE=1 SV=3 - [RIMB2_MOUSE]	1.33	0	↓
Q9CXW4	60S ribosomal protein L11 OS=Mus musculus GN=Rpl11 PE=1 SV=4 - [RL11_MOUSE]	1.00	0	↓
Q8BNW9	Kelch repeat and BTB domain-containing protein 11 OS=Mus musculus GN=Kbtbd11 PE=1 SV=3 - [KBTBB_MOUSE]	2.67	0.25	↓
P13634	Carbonic anhydrase 1 OS=Mus musculus GN=Ca1 PE=1 SV=4 - [CAH1_MOUSE]	1.00	8.5	↑
O70161	Phosphatidylinositol 4-phosphate 5-kinase type-1 gamma OS=Mus musculus GN=Pip5k1c PE=1 SV=2 - [PI51C_MOUSE]	7.00	2.75	↓
Q6IRU5	Clathrin light chain B OS=Mus musculus GN=Cltb PE=1 SV=1 - [CLCB_MOUSE]	3.00	2.25	↓
Q9ERR1	Nuclear distribution protein nudE-like 1 OS=Mus musculus GN=Ndel1 PE=1 SV=2 - [NDEL1_MOUSE]	1.33	1	↓
Q9QYB8	Beta-adducin OS=Mus musculus GN=Add2 PE=1 SV=4 - [ADDB_MOUSE]	12.33	8	↓
Q9D404	3-oxoacyl-[acyl-carrier-protein] synthase, mitochondrial OS=Mus musculus GN=Oxsm PE=1 SV=1 - [OXSM_MOUSE]	2.33	0.25	↓
P20918	Plasminogen OS=Mus musculus GN=Plg PE=1 SV=3 - [PLMN_MOUSE]	0.00	2	↑
O35927	Catenin delta-2 OS=Mus musculus GN=Ctnnd2 PE=1 SV=1 - [CTND2_MOUSE]	2.33	0.5	↓
Q3URE1	Acyl-CoA synthetase family member 3, mitochondrial OS=Mus musculus GN=Acsf3 PE=1 SV=2 - [ACSF3_MOUSE]	1.67	2.5	↑
P32067	Lupus La protein homolog OS=Mus musculus GN=Ssb PE=1 SV=1 - [LA_MOUSE]	2.00	0.5	↓
Q9CR21	Acyl carrier protein, mitochondrial OS=Mus musculus GN=Ndufab1 PE=1 SV=1 - [ACPM_MOUSE]	2.00	1.75	↓
Q9D7B6	Isobutyryl-CoA dehydrogenase, mitochondrial OS=Mus musculus GN=Acad8 PE=1 SV=2 - [ACAD8_MOUSE]	4.00	2	↓
Q9ERT9	Protein phosphatase 1 regulatory subunit 1A OS=Mus musculus GN=Ppp1r1a PE=1 SV=1 - [PPR1A_MOUSE]	1.33	0	↓
P84099	60S ribosomal protein L19 OS=Mus musculus GN=Rpl19 PE=1 SV=1 - [RL19_MOUSE]	1.00	0	↓
Q5DTT2	PH and SEC7 domain-containing protein 1 OS=Mus musculus GN=Psd PE=1 SV=2 - [PSD1_MOUSE]	1.00	0	↓
Q9JLB0	MAGUK p55 subfamily member 6 OS=Mus musculus GN=Mpp6 PE=1 SV=1 - [MPP6_MOUSE]	2.33	0.25	↓
P16330	2',3'-cyclic-nucleotide 3'-phosphodiesterase OS=Mus musculus GN=Cnp PE=1 SV=3 - [CN37_MOUSE]	19.33	15.5	↓
Q62188	Dihydropyrimidinase-related protein 3 OS=Mus musculus GN=Dpysl3 PE=1 SV=1 - [DPYL3_MOUSE]	15.33	14.75	↓
P28661	Septin-4 OS=Mus musculus GN=Sept4 PE=1 SV=1 - [SEPT4_MOUSE]	3.67	1.75	↓
P56135	ATP synthase subunit f, mitochondrial OS=Mus musculus GN=Atp5j2 PE=1 SV=3 - [ATPK_MOUSE]	1.67	1	↓
Q8C1B7	Septin-11 OS=Mus musculus GN=Sept11 PE=1 SV=4 - [SEP11_MOUSE]	10.67	7.75	↓
Q9Z2Q6	Septin-5 OS=Mus musculus GN=Sept5 PE=1 SV=2 - [SEPT5_MOUSE]	11.33	9.75	↓
B9EJA2	Cortactin-binding protein 2 OS=Mus musculus GN=Cttnbp2 PE=1 SV=2 - [CTTB2_MOUSE]	3.67	0.75	↓
Q8VCT3	Aminopeptidase B OS=Mus musculus GN=Rnpep PE=1 SV=2 - [AMPB_MOUSE]	5.33	1.25	↓
P07356	Annexin A2 OS=Mus musculus GN=Anxa2 PE=1 SV=2 - [ANXA2_MOUSE]	0.67	1.75	↑

Continued

Accession Number	Protein description	Sham	ICH	Brain injury-induced modulation Upregulation ↑ or Downregulation ↓
		Average number of total peptides	Average number of total peptides	
Q8BLQ9	Cell adhesion molecule 2 OS=Mus musculus GN=Cadm2 PE=1 SV=2 - [CADM2_MOUSE]	4.33	2.25	↓
Q8JZP2	Synapsin-3 OS=Mus musculus GN=Syn3 PE=1 SV=2 - [SYN3_MOUSE]	3.67	2.25	↓
P11352	Glutathione peroxidase 1 OS=Mus musculus GN=Gpx1 PE=1 SV=2 - [GPX1_MOUSE]	1.67	4.5	↑
Q64433	10 kDa heat shock protein, mitochondrial OS=Mus musculus GN=Hspe1 PE=1 SV=2 - [CH10_MOUSE]	5.33	5	↓
Q9QXM0	Abhydrolase domain-containing protein 2 OS=Mus musculus GN=Abhd2 PE=1 SV=1 - [ABHD2_MOUSE]	1.00	0	↓
Q812A2	SLIT-ROBO Rho GTPase-activating protein 3 OS=Mus musculus GN=Srgap3 PE=1 SV=1 - [SRGP3_MOUSE]	1.33	0	↓
P22682	E3 ubiquitin-protein ligase CBL OS=Mus musculus GN=Cbl PE=1 SV=3 - [CBL_MOUSE]	1.00	0	↓
Q80XN0	D-beta-hydroxybutyrate dehydrogenase, mitochondrial OS=Mus musculus GN=Bdh1 PE=1 SV=2 - [BDH_MOUSE]	1.33	0.5	↓
P06800	Receptor-type tyrosine-protein phosphatase C OS=Mus musculus GN=Ptprc PE=1 SV=3 - [PTPRC_MOUSE]	1.00	0	↓
Q9D2G2	Dihydrolipoyllysine-residue succinyltransferase component of 2-oxoglutarate dehydrogenase complex, mitochondrial OS=Mus musculus GN=Dlst PE=1 SV=1 - [ODO2_MOUSE]	6.33	3.75	↓
P49615	Cyclin-dependent-like kinase 5 OS=Mus musculus GN=Cdk5 PE=1 SV=1 - [CDK5_MOUSE]	5.00	1.25	↓
Q9CR62	Mitochondrial 2-oxoglutarate/malate carrier protein OS=Mus musculus GN=Slc25a11 PE=1 SV=3 - [M2OM_MOUSE]	8.33	5	↓
Q63810	Calcineurin subunit B type 1 OS=Mus musculus GN=Ppp3r1 PE=1 SV=3 - [CANB1_MOUSE]	9.00	9	↑
Q62418	Drebrin-like protein OS=Mus musculus GN=Dbnl PE=1 SV=2 - [DBNL_MOUSE]	5.00	2	↓
Q9CYK1	Tryptophan--tRNA ligase, mitochondrial OS=Mus musculus GN=Wars2 PE=1 SV=2 - [SYWM_MOUSE]	0.67	1	↑
P60840	Alpha-endosulfine OS=Mus musculus GN=Ensa PE=1 SV=1 - [ENSA_MOUSE]	1.67	0	↓
Q9CQE8	UPF0568 protein C14orf166 homolog OS=Mus musculus PE=1 SV=1 - [CN166_MOUSE]	2.67	0.75	↓
O88447	Kinesin light chain 1 OS=Mus musculus GN=Klc1 PE=1 SV=3 - [KLC1_MOUSE]	3.33	1	↓
Q99LB6	Methionine adenosyltransferase 2 subunit beta OS=Mus musculus GN=Mat2b PE=1 SV=1 - [MAT2B_MOUSE]	2.33	0.5	↓
P40240	CD9 antigen OS=Mus musculus GN=Cd9 PE=1 SV=2 - [CD9_MOUSE]	1.00	0	↓
P11031	Activated RNA polymerase II transcriptional coactivator p15 OS=Mus musculus GN=Sub1 PE=1 SV=3 - [TCP4_MOUSE]	1.00	0	↓
Q91WD5	NADH dehydrogenase [ubiquinone] iron-sulfur protein 2, mitochondrial OS=Mus musculus GN=Ndufs2 PE=1 SV=1 - [NDUS2_MOUSE]	5.00	2.5	↓
P07724	Serum albumin OS=Mus musculus GN=Alb PE=1 SV=3 - [ALBU_MOUSE]	17.00	26.75	↑
Q8K0E8	Fibrinogen beta chain OS=Mus musculus GN=Fgb PE=1 SV=1 - [FIBB_MOUSE]	0.00	10.75	↑
Q9CPQ8	ATP synthase subunit g, mitochondrial OS=Mus musculus GN=Atp5l PE=1 SV=1 - [ATP5L_MOUSE]	1.00	1.25	↓
Q9D1X0	Nucleolar protein 3 OS=Mus musculus GN=Nol3 PE=1 SV=1 - [NOL3_MOUSE]	2.67	0.25	↓
Q61490	CD166 antigen OS=Mus musculus GN=Alcam PE=1 SV=3 - [CD166_MOUSE]	3.00	0.75	↓
O88307	Sortilin-related receptor OS=Mus musculus GN=Sorl1 PE=1 SV=3 - [SORL_MOUSE]	1.33	0.33	↓

Continued

Accession Number	Protein description	Sham	ICH	Brain injury-induced modulation Upregulation ↑ or Downregulation ↓
		Average number of total peptides	Average number of total peptides	
Q8BLK3	Limbic system-associated membrane protein OS=Mus musculus GN=Lsamp PE=1 SV=1 - [LSAMP_MOUSE]	4.00	3.25	↓
P0C6F1	Dynein heavy chain 2, axonemal OS=Mus musculus GN=Dnah2 PE=1 SV=1 - [DYH2_MOUSE]	2.00	0.5	↓
Q8K3J1	NADH dehydrogenase [ubiquinone] iron-sulfur protein 8, mitochondrial OS=Mus musculus GN=Ndufs8 PE=1 SV=1 - [NDUS8_MOUSE]	2.33	1.3	↓
Q03734	Serine protease inhibitor A3M OS=Mus musculus GN=Serpina3m PE=1 SV=2 - [SPA3M_MOUSE]	0.00	5.75	↑
Q60973	Histone-binding protein RBBP7 OS=Mus musculus GN=Rbbp7 PE=1 SV=1 - [RBBP7_MOUSE]	3.33	0.5	↓
P31725	Protein S100-A9 OS=Mus musculus GN=S100a9 PE=1 SV=3 - [S10A9_MOUSE]	0.00	1.75	↑
Q91X97	Neurocalcin-delta OS=Mus musculus GN=Ncald PE=1 SV=4 - [NCALD_MOUSE]	5.00	4.25	↓
P70296	Phosphatidylethanolamine-binding protein 1 OS=Mus musculus GN=Pebp1 PE=1 SV=3 - [PEBP1_MOUSE]	8.67	8.25	↓
O88643	Serine/threonine-protein kinase PAK 1 OS=Mus musculus GN=Pak1 PE=1 SV=1 - [PAK1_MOUSE]	7.00	3.25	↓
Q5SQX6	Cytoplasmic FMR1-interacting protein 2 OS=Mus musculus GN=Cyfip2 PE=1 SV=2 - [CYFP2_MOUSE]	7.00	3	↓
Q71RI9	Kynurenine--oxoglutarate transaminase 3 OS=Mus musculus GN=Ccbl2 PE=1 SV=1 - [KAT3_MOUSE]	2.67	0	↓
Q921I1	Serotransferrin OS=Mus musculus GN=Tf PE=1 SV=1 - [TRFE_MOUSE]	5.33	20.25	↑
Q9CZ13	Cytochrome b-c1 complex subunit 1, mitochondrial OS=Mus musculus GN=Uqcrc1 PE=1 SV=2 - [QCR1_MOUSE]	10.33	9	↓
Q8R4N0	Citrate lyase subunit beta-like protein, mitochondrial OS=Mus musculus GN=Clybl PE=1 SV=2 - [CLYBL_MOUSE]	4.00	1.5	↓
O08677	Kininogen-1 OS=Mus musculus GN=Kng1 PE=1 SV=1 - [KNG1_MOUSE]	0.00	1.5	↑
Q60972	Histone-binding protein RBBP4 OS=Mus musculus GN=Rbbp4 PE=1 SV=5 - [RBBP4_MOUSE]	3.67	0.75	↓
Q0VE82	Copine-7 OS=Mus musculus GN=Cpne7 PE=1 SV=1 - [CPNE7_MOUSE]	1.00	1.25	↑
P29391	Ferritin light chain 1 OS=Mus musculus GN=Ftl1 PE=1 SV=2 - [FRIL1_MOUSE]	2.00	5.5	↑
Q64522	Histone H2A type 2-B OS=Mus musculus GN=Hist2h2ab PE=1 SV=3 - [H2A2B_MOUSE]	3.00	3	↑
Q9Z2I8	Succinyl-CoA ligase [GDP-forming] subunit beta, mitochondrial OS=Mus musculus GN=Suclg2 PE=1 SV=3 - [SUCB2_MOUSE]	5.67	1.5	↓
O88712	C-terminal-binding protein 1 OS=Mus musculus GN=Ctbp1 PE=1 SV=2 - [CTBP1_MOUSE]	5.33	3.5	↓
Q00896	Alpha-1-antitrypsin 1-3 OS=Mus musculus GN=Serpina1c PE=1 SV=2 - [A1AT3_MOUSE]	0.33	8.25	↑
P35762	CD81 antigen OS=Mus musculus GN=Cd81 PE=1 SV=2 - [CD81_MOUSE]	3.00	2.75	↓
Q9JKC6	Cell cycle exit and neuronal differentiation protein 1 OS=Mus musculus GN=Cend1 PE=1 SV=1 - [CEND_MOUSE]	1.00	0	↓
Q91VR2	ATP synthase subunit gamma, mitochondrial OS=Mus musculus GN=Atp5c1 PE=1 SV=1 - [ATPG_MOUSE]	7.00	6.25	↓
O08539	Myc box-dependent-interacting protein 1 OS=Mus musculus GN=Bin1 PE=1 SV=1 - [BIN1_MOUSE]	11.33	8.5	↓
Q99LX0	Protein deglycase DJ-1 OS=Mus musculus GN=Park7 PE=1 SV=1 - [PARK7_MOUSE]	7.33	4.5	↓
Q9JII6	Alcohol dehydrogenase [NADP(+)] OS=Mus musculus GN=Akr1a1 PE=1 SV=3 - [AK1A1_MOUSE]	5.00	3	↓
O08532	Voltage-dependent calcium channel subunit alpha-2/delta-1 OS=Mus musculus GN=Cacna2d1 PE=1 SV=1 - [CA2D1_MOUSE]	6.33	2.5	↓

Continued

Accession Number	Protein description	Sham	ICH	Brain injury-induced modulation Upregulation ↑ or Downregulation ↓
		Average number of total peptides	Average number of total peptides	
Q71M36	Chondroitin sulfate proteoglycan 5 OS=Mus musculus GN=Cspg5 PE=1 SV=2 - [CSPG5_MOUSE]	2.67	0.75	↓
P02104	Hemoglobin subunit epsilon-Y2 OS=Mus musculus GN=Hbb-y PE=1 SV=2 - [HBE_MOUSE]	1.00	1.75	↑
Q3THE2	Myosin regulatory light chain 12B OS=Mus musculus GN=Myl12b PE=1 SV=2 - [ML12B_MOUSE]	4.67	2.75	↓
Q3ULJ0	Glycerol-3-phosphate dehydrogenase 1-like protein OS=Mus musculus GN=Gpd1l PE=1 SV=2 - [GPD1L_MOUSE]	8.00	4	↓
Q8CHG3	GRIP and coiled-coil domain-containing protein 2 OS=Mus musculus GN=Gcc2 PE=1 SV=2 - [GCC2_MOUSE]	0.00	1.25	↑
P21614	Vitamin D-binding protein OS=Mus musculus GN=Gc PE=1 SV=2 - [VTDB_MOUSE]	0.00	2.5	↑
P62748	Hippocalcin-like protein 1 OS=Mus musculus GN=Hpcal1 PE=1 SV=2 - [HPCL1_MOUSE]	4.33	3.75	↓
P35803	Neuronal membrane glycoprotein M6-b OS=Mus musculus GN=Gpm6b PE=1 SV=2 - [GPM6B_MOUSE]	3.67	2.75	↓
O70318	Band 4.1-like protein 2 OS=Mus musculus GN=Epb41l2 PE=1 SV=2 - [E41L2_MOUSE]	5.33	2.75	↓
Q8BH04	Phosphoenolpyruvate carboxykinase [GTP], mitochondrial OS=Mus musculus GN=Pck2 PE=1 SV=1 - [PCKGM_MOUSE]	3.67	0	↓
O88998	Noelin OS=Mus musculus GN=Olfm1 PE=1 SV=1 - [NOE1_MOUSE]	1.67	0	↓
Q9JHK4	Geranylgeranyl transferase type-2 subunit alpha OS=Mus musculus GN=Rabggta PE=1 SV=1 - [PGTA_MOUSE]	3.00	0.75	↓
Q61838	Alpha-2-macroglobulin OS=Mus musculus GN=A2m PE=1 SV=3 - [A2M_MOUSE]	0.00	14	↑
P01942	Hemoglobin subunit alpha OS=Mus musculus GN=Hba PE=1 SV=2 - [HBA_MOUSE]	6.00	8	↑
P28650	Adenylosuccinate synthetase isozyme 1 OS=Mus musculus GN=Adssl1 PE=1 SV=2 - [PURA1_MOUSE]	3.00	1.25	↓
P04370	Myelin basic protein OS=Mus musculus GN=Mbp PE=1 SV=2 - [MBP_MOUSE]	3.00	2.75	↓
P16858	Glyceraldehyde-3-phosphate dehydrogenase OS=Mus musculus GN=Gapdh PE=1 SV=2 - [G3P_MOUSE]	13.33	13.75	↓
Q9D023	Mitochondrial pyruvate carrier 2 OS=Mus musculus GN=Mpc2 PE=1 SV=1 - [MPC2_MOUSE]	1.33	0.25	↓
P62889	60S ribosomal protein L30 OS=Mus musculus GN=Rpl30 PE=1 SV=2 - [RL30_MOUSE]	1.67	2	↑
Q00623	Apolipoprotein A-I OS=Mus musculus GN=Apoa1 PE=1 SV=2 - [APOA1_MOUSE]	0.00	2.75	↑
P03911	NADH-ubiquinone oxidoreductase chain 4 OS=Mus musculus GN=Mtnd4 PE=1 SV=1 - [NU4M_MOUSE]	1.33	0.75	↓
Q9QUR6	Prolyl endopeptidase OS=Mus musculus GN=Prep PE=1 SV=1 - [PPCE_MOUSE]	3.67	1.75	↓
Q61792	LIM and SH3 domain protein 1 OS=Mus musculus GN=Lasp1 PE=1 SV=1 - [LASP1_MOUSE]	1.67	2	↑
Q91W43	Glycine dehydrogenase (decarboxylating), mitochondrial OS=Mus musculus GN=Gldc PE=1 SV=1 - [GCSP_MOUSE]	1.67	0.25	↓
P07759	Serine protease inhibitor A3K OS=Mus musculus GN=Serpina3k PE=1 SV=2 - [SPA3K_MOUSE]	1.00	13.75	↑
P62761	Visinin-like protein 1 OS=Mus musculus GN=Vsnl1 PE=1 SV=2 - [VISL1_MOUSE]	6.33	4.5	↓
F6ZDS4	Nucleoprotein TPR OS=Mus musculus GN=Tpr PE=1 SV=1 - [TPR_MOUSE]	3.00	0.5	↓
Q8CBE3	WD repeat-containing protein 37 OS=Mus musculus GN=Wdr37 PE=1 SV=1 - [WDR37_MOUSE]	1.67	0.5	↓
P47791	Glutathione reductase, mitochondrial OS=Mus musculus GN=Gsr PE=1 SV=3 - [GSHR_MOUSE]	7.33	3.5	↓
Q62059	Versican core protein OS=Mus musculus GN=Vcan PE=1 SV=2 - [CSPG2_MOUSE]	8.33	4.25	↓
P84075	Neuron-specific calcium-binding protein hippocalcin OS=Mus musculus GN=Hpca PE=1 SV=2 - [HPCA_MOUSE]	7.33	7	↓

Continued

Accession Number	Protein description	Sham	ICH	Brain injury-induced modulation Upregulation ↑ or Downregulation ↓
		Average number of total peptides	Average number of total peptides	
Q80XI4	Phosphatidylinositol 5-phosphate 4-kinase type-2 beta OS=Mus musculus GN=Pip4k2b PE=1 SV=1 - [PI42B_MOUSE]	2.67	1.66	↓
P01027	Complement C3 OS=Mus musculus GN=C3 PE=1 SV=3 - [CO3_MOUSE]	1.00	9.5	↑
O88935	Synapsin-1 OS=Mus musculus GN=Syn1 PE=1 SV=2 - [SYN1_MOUSE]	21.00	19.25	↓
Q9QUR7	Peptidyl-prolyl cis-trans isomerase NIMA-interacting 1 OS=Mus musculus GN=Pin1 PE=1 SV=1 - [PIN1_MOUSE]	3.00	1.25	↓
Q9CQC7	NADH dehydrogenase [ubiquinone] 1 beta subcomplex subunit 4 OS=Mus musculus GN=Ndufb4 PE=1 SV=3 - [NDUB4_MOUSE]	1.33	0.25	↓
P21958	Antigen peptide transporter 1 OS=Mus musculus GN=Tap1 PE=1 SV=3 - [TAP1_MOUSE]	1.33	0.25	↓
Q8VEM8	Phosphate carrier protein, mitochondrial OS=Mus musculus GN=Slc25a3 PE=1 SV=1 - [MPCP_MOUSE]	5.00	3	↓
P22599	Alpha-1-antitrypsin 1-2 OS=Mus musculus GN=Serpina1b PE=1 SV=2 - [A1AT2_MOUSE]	1.00	5.5	↑
Q9Z1S5	Neuronal-specific septin-3 OS=Mus musculus GN=Sept3 PE=1 SV=2 - [SEPT3_MOUSE]	6.00	4.75	↓
P57780	Alpha-actinin-4 OS=Mus musculus GN=Actn4 PE=1 SV=1 - [ACTN4_MOUSE]	20.00	11.5	↓
Q923D2	Flavin reductase (NADPH) OS=Mus musculus GN=Blvrb PE=1 SV=3 - [BLVRB_MOUSE]	2.67	5.75	↑
O88952	Protein lin-7 homolog C OS=Mus musculus GN=Lin7c PE=1 SV=2 - [LIN7C_MOUSE]	2.67	1	↓
P02088	Hemoglobin subunit beta-1 OS=Mus musculus GN=Hbb-b1 PE=1 SV=2 - [HBB1_MOUSE]	11.33	15	↑
Q9R0Q6	Actin-related protein 2/3 complex subunit 1A OS=Mus musculus GN=Arpc1a PE=1 SV=1 - [ARC1A_MOUSE]	10.00	8.75	↓
P63158	High mobility group protein B1 OS=Mus musculus GN=Hmgb1 PE=1 SV=2 - [HMGB1_MOUSE]	0.33	1	↑
P99028	Cytochrome b-c1 complex subunit 6, mitochondrial OS=Mus musculus GN=Uqcrh PE=1 SV=2 - [QCR6_MOUSE]	1.00	1	↓
Q9CWZ7	Gamma-soluble NSF attachment protein OS=Mus musculus GN=Napg PE=1 SV=1 - [SNAG_MOUSE]	6.33	3.5	↓
Q9CWS0	N(G),N(G)-dimethylarginine dimethylaminohydrolase 1 OS=Mus musculus GN=Ddah1 PE=1 SV=3 - [DDAH1_MOUSE]	7.00	2.75	↓
P52503	NADH dehydrogenase [ubiquinone] iron-sulfur protein 6, mitochondrial OS=Mus musculus GN=Ndufs6 PE=1 SV=2 - [NDUS6_MOUSE]	1.67	0.25	↓
P47857	ATP-dependent 6-phosphofructokinase, muscle type OS=Mus musculus GN=Pfkm PE=1 SV=3 - [PFKAM_MOUSE]	22.67	20.25	↓
P10639	Thioredoxin OS=Mus musculus GN=Txn PE=1 SV=3 - [THIO_MOUSE]	2.67	3.25	↑
Q91V09	WD repeat-containing protein 13 OS=Mus musculus GN=Wdr13 PE=1 SV=1 - [WDR13_MOUSE]	1.67	0.5	↓
P62137	Serine/threonine-protein phosphatase PP1-alpha catalytic subunit OS=Mus musculus GN=Ppp1ca PE=1 SV=1 - [PP1A_MOUSE]	13.33	9	↓
Q9ERG2	Striatin-3 OS=Mus musculus GN=Strn3 PE=1 SV=1 - [STRN3_MOUSE]	2.00	0.5	↓
Q60864	Stress-induced-phosphoprotein 1 OS=Mus musculus GN=Stip1 PE=1 SV=1 - [STIP1_MOUSE]	11.00	7.25	↓
Q00897	Alpha-1-antitrypsin 1-4 OS=Mus musculus GN=Serpina1d PE=1 SV=1 - [A1AT4_MOUSE]	0.33	5	↑
Q9DCS3	Trans-2-enoyl-CoA reductase, mitochondrial OS=Mus musculus GN=Mecr PE=1 SV=2 - [MECR_MOUSE]	1.67	0.25	↓
P47754	F-actin-capping protein subunit alpha-2 OS=Mus musculus GN=Capza2 PE=1 SV=3 - [CAZA2_MOUSE]	9.33	9.25	↑
O70435	Proteasome subunit alpha type-3 OS=Mus musculus GN=Psma3 PE=1 SV=3 - [PSA3_MOUSE]	3.00	1.75	↓
Q6P3A8	2-oxoisovalerate dehydrogenase subunit beta, mitochondrial OS=Mus musculus GN=Bckdhb PE=1 SV=2 - [ODBB_MOUSE]	1.33	2	↑

Continued

Accession Number	Protein description	Sham	ICH	Brain injury-induced modulation Upregulation ↑ or Downregulation ↓
		Average number of total peptides	Average number of total peptides	
Q9Z0P5	Twinfilin-2 OS=Mus musculus GN=Twf2 PE=1 SV=1 - [TWF2_MOUSE]	4.00	1.5	↓
Q9JJU8	SH3 domain-binding glutamic acid-rich-like protein OS=Mus musculus GN=Sh3bgrl PE=1 SV=1 - [SH3L1_MOUSE]	3.33	1	↓
Q91WP6	Serine protease inhibitor A3N OS=Mus musculus GN=Serpina3n PE=1 SV=1 - [SPA3N_MOUSE]	0.00	2	↑
O35625	Axin-1 OS=Mus musculus GN=Axin1 PE=1 SV=3 - [AXIN1_MOUSE]	1.00	0.25	↓
Q61885	Myelin-oligodendrocyte glycoprotein OS=Mus musculus GN=Mog PE=1 SV=1 - [MOG_MOUSE]	1.33	1.5	↑
Q9DC69	NADH dehydrogenase [ubiquinone] 1 alpha subcomplex subunit 9, mitochondrial OS=Mus musculus GN=Ndufa9 PE=1 SV=2 - [NDUA9_MOUSE]	4.33	2.25	↓
P14901	Heme oxygenase 1 OS=Mus musculus GN=Hmox1 PE=1 SV=1 - [HMOX1_MOUSE]	0.00	2.25	↑
Q9CVB6	Actin-related protein 2/3 complex subunit 2 OS=Mus musculus GN=Arpc2 PE=1 SV=3 - [ARPC2_MOUSE]	10.00	5.75	↓
Q9WVJ2	26S proteasome non-ATPase regulatory subunit 13 OS=Mus musculus GN=Psmd13 PE=1 SV=1 - [PSD13_MOUSE]	0.33	1	↑
Q8BYR5	Calcium-dependent secretion activator 2 OS=Mus musculus GN=Cadps2 PE=1 SV=2 - [CAPS2_MOUSE]	5.33	3	↓
Q9CR68	Cytochrome b-c1 complex subunit Rieske, mitochondrial OS=Mus musculus GN=Uqcrfs1 PE=1 SV=1 - [UCRI_MOUSE]	5.00	3.5	↓
Q9DB20	ATP synthase subunit O, mitochondrial OS=Mus musculus GN=Atp5o PE=1 SV=1 - [ATPO_MOUSE]	7.00	5.5	↓
Q9R0P3	S-formylglutathione hydrolase OS=Mus musculus GN=Esd PE=1 SV=1 - [ESTD_MOUSE]	5.00	4.25	↓
P61982	14-3-3 protein gamma OS=Mus musculus GN=Ywhag PE=1 SV=2 - [1433G_MOUSE]	8.33	8	↓
Q9CQJ8	NADH dehydrogenase [ubiquinone] 1 beta subcomplex subunit 9 OS=Mus musculus GN=Ndufb9 PE=1 SV=3 - [NDUB9_MOUSE]	2.00	0.25	↓
Q62420	Endophilin-A1 OS=Mus musculus GN=Sh3gl2 PE=1 SV=2 - [SH3G2_MOUSE]	10.67	8.25	↓
O35215	D-dopachrome decarboxylase OS=Mus musculus GN=Ddt PE=1 SV=3 - [DOPD_MOUSE]	4.33	2.75	↓
Q99JY9	Actin-related protein 3 OS=Mus musculus GN=Actr3 PE=1 SV=3 - [ARP3_MOUSE]	14.33	10.75	↓
P54227	Stathmin OS=Mus musculus GN=Stmn1 PE=1 SV=2 - [STMN1_MOUSE]	4.00	3.5	↓
Q61831	Mitogen-activated protein kinase 10 OS=Mus musculus GN=Mapk10 PE=1 SV=2 - [MK10_MOUSE]	2.67	1.25	↓
Q9QXS6	Drebrin OS=Mus musculus GN=Dbn1 PE=1 SV=4 - [DREB_MOUSE]	13.33	9.75	↓
Q61704	Inter-alpha-trypsin inhibitor heavy chain H3 OS=Mus musculus GN=Itih3 PE=1 SV=3 - [ITIH3_MOUSE]	0.33	3	↑
P62242	40S ribosomal protein S8 OS=Mus musculus GN=Rps8 PE=1 SV=2 - [RS8_MOUSE]	1.67	2.5	↑
Q9D8Y0	EF-hand domain-containing protein D2 OS=Mus musculus GN=Efhd2 PE=1 SV=1 - [EFHD2_MOUSE]	8.00	7	↓
Q8K0S0	Phytanoyl-CoA hydroxylase-interacting protein OS=Mus musculus GN=Phyhip PE=1 SV=1 - [PHYIP_MOUSE]	7.67	4	↓
Q9DCU6	39S ribosomal protein L4, mitochondrial OS=Mus musculus GN=Mrpl4 PE=1 SV=1 - [RM04_MOUSE]	1.00	1	↑
Q99MR8	Methylcrotonoyl-CoA carboxylase subunit alpha, mitochondrial OS=Mus musculus GN=Mccc1 PE=1 SV=2 - [MCCA_MOUSE]	4.67	0.75	↓
P56212	cAMP-regulated phosphoprotein 19 OS=Mus musculus GN=Arpp19 PE=1 SV=2 - [ARP19_MOUSE]	2.67	0.75	↓
