# Supplementary material for: A Combined Proteomics and Bioinformatics Approach Reveals Novel Signaling Pathways and Molecular Targets After Intracerebral Hemorrhage
Source: J Mol Neurosci. 2020 Mar 13;70(8):1186–97. doi: 10.1007/s12031-020-01526-7 (PMC7359136; doi:10.1007/s12031-020-01526-7)
Supplement: Supplementary file 2 — (DOCX 23 kb) [file 12031_2020_1526_MOESM2_ESM.docx]

**Supplementary data. Table 2**

| **Protein class** | **Protein name** |
| --- | --- |
| **Calcium binding protein** |  |
|  | Serine/threonine-protein phosphatase PP1-alpha catalytic subunit |
|  | Myosin regulatory light chain 12B |
|  | Protein S100-A9 |
|  | Calcium-dependent secretion activator 2 |
|  | EF-hand domain-containing protein D2 |
|  | Calpain-8 |
| **Cell adhesion molecule** |  |
|  | Protein lin-7 homolog C |
|  | CD166 antigen |
| **Cell junction protein** |  |
|  | Protein lin-7 homolog C |
|  | MAGUK p55 subfamily member 6 |
| **Chaperone** |  |
|  | 10 k Da heat shock protein, mitochondrial |
|  | 14-3-3 protein gamma |
| **Cytoskeletal protein** |  |
|  | Septin-4 |
|  | Beta-adducin |
|  | Actin-related protein 2/3 complex subunit 1A |
|  | Drebrin-like protein |
|  | Myosin regulatory light chain 12B |
|  | Cytoskeleton-associated protein 5 |
|  | Drebrin |
|  | Catenin delta-2 |
|  | Synapsin-1 |
|  | Actin-related protein 3 |
|  | Synapsin-3 |
|  | Twinfilin-2 |
|  | Neuronal-specific septin-3 |
|  | F-actin-capping protein subunit alpha-2 |
|  | Dynein heavy chain 2, axonemal |
|  | Septin-11 |
|  | Protein kinase C and casein kinase substrate in neurons protein 1 |
|  | Septin-5 |
| **Defense/Immunity protein** |  |
|  | Complement C3 |
|  | Alpha-2-macroglobulin |
| **Enzyme modulator** |  |
|  | SLIT-ROBO Rho GTPase-activating protein 3 |
|  | Septin-4 |
|  | Alpha-1-antitrypsin 1-4 |
|  | Alpha-1-antitrypsin 1-2 |
|  | Serine protease inhibitor A3N |
|  | Complement C3 |
|  | Serine protease inhibitor A3K |
|  | Protein phosphatase 1 regulatory subunit 1A |
|  | Kininogen-1 |
|  | Serine protease inhibitor A3M |
|  | Alpha-1-antitrypsin 1-3 |
|  | Phosphatidylethanolamine-binding protein 1 |
|  | Neuronal-specific septin-3 |
|  | 26S proteasome non-ATPase regulatory subunit 13 |
|  | Alpha-2-macroglobulin |
|  | Cytoplasmic FMR1-interacting protein 2 |
|  | Septin-11 |
|  | Myelin-oligodendrocyte glycoprotein |
|  | Septin-5 |
|  | Inter-alpha-trypsin inhibitor heavy chain H3 |
| **Extracellular matrix protein** |  |
|  | Versican core protein |
| **Hydrolase** |  |
|  | S-formylglutathione hydrolase |
|  | Serine/threonine-protein phosphatase PP1-alpha catalytic subunit |
|  | Lipase member K |
|  | Monoacylglycerol lipase ABHD2 |
|  | Antigen peptide transporter 1 |
|  | Dihydropyrimidinase-related protein 3 |
|  | Plasminogen |
|  | Prolyl endopeptidase |
|  | Receptor-type tyrosine-protein phosphatase C |
|  | ATP synthase subunit O, mitochondrial |
|  | Calpain-8 |
|  | Protein/nucleic acid deglycase DJ-1 |
|  | Serotransferrin |
|  | Cytochrome b-c1 complex subunit 1, mitochondrial |
|  | Dynein heavy chain 2, axonemal |
|  | N(G), N(G)-dimethylarginine dimethylaminohydrolase 1 |
|  | 2',3'-cyclic-nucleotide 3'-phosphodiesterase |
| **Isomerase** |  |
|  | D-dopachrome decarboxylase |
| **Ligase** |  |
|  | E3 ubiquitin-protein ligase CBL |
|  | Succinate--CoA ligase [GDP-forming] subunit beta, mitochondrial |
|  | Adenylosuccinate synthetase isozyme 1 |
|  | Methylcrotonoyl-CoA carboxylase subunit alpha, mitochondrial |
|  | Acyl-CoA synthetase family member 3, mitochondrial |
|  | Asparagine--tRNA ligase, cytoplasmic |
| **Lyase** |  |
|  | 2-oxoisovalerate dehydrogenase subunit beta, mitochondrial |
|  | Phosphoenolpyruvate carboxykinase [GTP], mitochondrial |
|  | Cytoplasmic aconitate hydratase |
| **Membrane traffic protein** |  |
|  | Synaptosomal-associated protein 25 |
|  | Synapsin-1 |
|  | Clathrin light chain B |
|  | Synapsin-3 |
|  | Protein kinase C and casein kinase substrate in neurons protein 1 |
| **Nucleic acid binding** |  |
|  | Histone H1.5 |
|  | Lupus La protein homolog |
|  | Capping protein, Arp2/3 and myosin-I linker protein 2 |
|  | High mobility group protein B1 |
|  | 60S ribosomal protein L11 |
|  | 60S ribosomal protein L30 |
|  | Histone H2A type 2-B |
|  | Protein/nucleic acid deglycase DJ-1 |
|  | 60S ribosomal protein L19 |
|  | Asparagine--tRNA ligase, cytoplasmic |
| **Oxidoreductase** |  |
|  | Glycine dehydrogenase (decarboxylating), mitochondrial |
|  | Glyceraldehyde-3-phosphate dehydrogenase |
|  | NADH-ubiquinone oxidoreductase 75 k Da subunit, mitochondrial |
|  | Cytochrome c oxidase subunit 5B, mitochondrial |
|  | NADH dehydrogenase [ubiquinone] iron-sulfur protein 6, mitochondrial |
|  | NADH dehydrogenase [ubiquinone] iron-sulfur protein 2, mitochondrial |
|  | Glutathione peroxidase 1 |
|  | Alcohol dehydrogenase [NADP (+)] |
|  | Glutathione reductase, mitochondrial |
|  | Flavin reductase (NADPH) |
|  | Cytochrome b-c1 complex subunit 6, mitochondrial |
|  | NADH dehydrogenase [ubiquinone] 1 alpha subcomplex subunit 9, mitochondrial |
|  | D-beta-hydroxybutyrate dehydrogenase, mitochondrial |
|  | Glycerol-3-phosphate dehydrogenase 1-like protein |
|  | 2-oxoisovalerate dehydrogenase subunit beta, mitochondrial |
|  | NADH dehydrogenase [ubiquinone] 1 beta subcomplex subunit 9 |
|  | NADH-ubiquinone oxidoreductase chain 4 |
|  | Isobutyryl-CoA dehydrogenase, mitochondrial |
|  | Cytochrome b-c1 complex subunit 7 |
|  | NADH dehydrogenase [ubiquinone] iron-sulfur protein 8, mitochondrial |
|  | NADH dehydrogenase [ubiquinone] 1 beta subcomplex subunit 4 |
| **Receptor** |  |
|  | Noelin |
|  | CD166 antigen |
|  | Sortilin-related receptor |
|  | Receptor-type tyrosine-protein phosphatase C |
|  | Serotransferrin |
| **Signaling molecule** |  |
|  | Fibrinogen gamma chain |
|  | High mobility group protein B1 |
|  | Protein S100-A9 |
|  | Complement C3 |
|  | Protein phosphatase 1 regulatory subunit 1A |
|  | D-dopachrome decarboxylase |
|  | Fibrinogen beta chain |
|  | Alpha-2-macroglobulin |
| **Storage protein** |  |
|  | Ferritin light chain 1 |
| **Structural protein** |  |
|  | Noelin |
|  | Neuronal membrane glycoprotein M6-b |
| **Transcription factor** |  |
|  | C-terminal-binding protein 1 |
|  | Capping protein, Arp2/3 and myosin-I linker protein 2 |
|  | High mobility group protein B1 |
|  | Protein/nucleic acid deglycase DJ-1 |
| **Transfer/carrier protein** |  |
|  | Serum albumin |
|  | Acyl carrier protein, mitochondrial |
|  | Vitamin D-binding protein |
|  | Serotransferrin |
| **Transferase** |  |
|  | ATP-dependent 6-phosphofructokinase, platelet type |
|  | Phosphatidylinositol 4-phosphate 5-kinase type-1 gamma |
|  | ATP-dependent 6-phosphofructokinase, muscle type |
|  | Kynurenine--oxoglutarate transaminase 3 |
|  | Phosphatidylinositol 5-phosphate 4-kinase type-2 beta |
|  | Geranylgeranyl transferase type-2 subunit alpha |
|  | Dihydrolipoyllysine-residue succinyltransferase component of 2-oxoglutarate dehydrogenase complex, mitochondrial |
|  | 2-oxoisovalerate dehydrogenase subunit beta, mitochondrial |
|  | Cyclin-dependent-like kinase 5 |
|  | Mitogen-activated protein kinase 10 |
|  | MAGUK p55 subfamily member 6 |
|  | Isobutyryl-CoA dehydrogenase, mitochondrial |
|  | Alanine aminotransferase 1 |
| **Transporter** |  |
|  | Sortilin-related receptor |
|  | ATP synthase subunit O, mitochondrial |
|  | Electroneutral sodium bicarbonate exchanger 1 |
|  | ATP synthase subunit f, mitochondrial |
