# Supplementary material for: A Combined Proteomics and Bioinformatics Approach Reveals Novel Signaling Pathways and Molecular Targets After Intracerebral Hemorrhage
Source: J Mol Neurosci. 2020 Mar 13;70(8):1186–97. doi: 10.1007/s12031-020-01526-7 (PMC7359136; doi:10.1007/s12031-020-01526-7)
Supplement: Supplementary file 3 — (DOCX 22 kb) [file 12031_2020_1526_MOESM3_ESM.docx]

**Supplementary data. Table 3**

| **S. No** | **Name of the pathway** | **Percent of protein hit against total number of proteins** |
| --- | --- | --- |
|  | Beta3 adrenergic receptor signaling pathway | 0.5% |
|  | Metabotropic glutamate receptor group III pathway | 0.5% |
|  | Beta2 adrenergic receptor signaling pathway | 0.5% |
|  | Axon guidance mediated by semaphorins | 1.4% |
|  | JAK/STAT signaling pathway | 0.5% |
|  | Beta1 adrenergic receptor signaling pathway | 0.5% |
|  | Apoptosis signaling pathway | 0.5% |
|  | 5HT4 type receptor mediated signaling pathway | 0.5% |
|  | De novo purine biosynthesis | 0.5% |
|  | Angiogenesis | 1.9% |
|  | Ionotropic glutamate receptor pathway | 0.5% |
|  | 5HT3 type receptor mediated signaling pathway | 0.5% |
|  | 5HT2 type receptor mediated signaling pathway | 0.5% |
|  | Alzheimer disease-amyloid secretase pathway | 1.0% |
|  | Interferon-gamma signaling pathway | 0.5% |
|  | 5HT1 type receptor mediated signaling pathway | 0.5% |
|  | Alpha adrenergic receptor signaling pathway | 0.5% |
|  | Integrin signalling pathway | 1.9% |
|  | Adrenaline and noradrenaline biosynthesis | 0.5% |
|  | Inflammation mediated by chemokine and cytokine signaling pathway | 1.4% |
|  | Hypoxia response via HIF activation | 0.5% |
|  | Ubiquitin proteasome pathway | 0.5% |
|  | Nicotine pharmacodynamics pathway | 1.4% |
|  | Synaptic vesicle trafficking | 1.0% |
|  | Huntington disease | 2.9% |
|  | p53 pathway | 1.9% |
|  | p53 pathway feedback loops 2 | 1.0% |
|  | Heterotrimeric G-protein signaling pathway-Gq alpha and Go alpha mediated pathway | 0.5% |
|  | p53 pathway by glucose deprivation | 1.0% |
|  | Heterotrimeric G-protein signaling pathway-Gi alpha and Gs alpha mediated pathway | 0.5% |
|  | Wnt signaling pathway | 2.9% |
|  | Vitamin D metabolism and pathway | 0.5% |
|  | Vasopressin synthesis | 0.5% |
|  | O-antigen biosynthesis | 0.5% |
|  | Glycolysis | 1.0% |
|  | Thyrotropin-releasing hormone receptor signaling pathway | 0.5% |
|  | Toll receptor signaling pathway | 0.5% |
|  | Ras Pathway | 1.0% |
|  | T cell activation | 1.9% |
|  | Methylcitrate cycle | 0.5% |
|  | FGF signaling pathway | 2.4% |
|  | TGF-beta signaling pathway | 0.5% |
|  | Oxytocin receptor mediated signaling pathway | 0.5% |
|  | FAS signaling pathway | 0.5% |
|  | ATP synthesis | 0.5% |
|  | Plasminogen activating cascade | 1.9% |
|  | EGF receptor signaling pathway | 2.9% |
|  | Parkinson disease | 3.4% |
|  | Opioid proopiomelanocortin pathway | 0.5% |
|  | Cytoskeletal regulation by Rho GTPase | 1.9% |
|  | PDGF signaling pathway | 1.4% |
|  | Opioid prodynorphin pathway | 0.5% |
|  | Oxidative stress response | 1.0% |
|  | Opioid proenkephalin pathway | 0.5% |
|  | Cell cycle | 0.5% |
|  | Nicotinic acetylcholine receptor signaling pathway | 1.0% |
|  | Cadherin signaling pathway | 0.5% |
|  | Muscarinic acetylcholine receptor 2 and 4 signaling pathway | 1.0% |
|  | Blood coagulation | 4.3% |
|  | Dopamine receptor mediated signaling pathway | 1.9% |
|  | Muscarinic acetylcholine receptor 1 and 3 signaling pathway | 0.5% |
|  | B cell activation | 1.0% |
|  | Cortocotropin releasing factor receptor signaling pathway | 0.5% |
|  | Metabotropic glutamate receptor group II pathway | 0.5% |
|  | CCKR signaling map | 1.0% |
|  | Pyruvate metabolism | 0.5% |
|  | Pyrimidine Metabolism | 0.5% |
